# Patterns and drivers of microbiome in different rock surface soil under the volcanic extreme environment

**DOI:** 10.1002/imt2.122

**Published:** 2023-06-19

**Authors:** Jin Chen, Zishan Li, Daolong Xu, Qingchen Xiao, Haijing Liu, Xiaoyu Li, Lumeng Chao, Hanting Qu, Yaxin Zheng, Xinyan Liu, Pengfei Wang, Yuying Bao

**Affiliations:** ^1^ Key Laboratory of Forage and Endemic Crop Biotechnology, Ministry of Education, School of Life Sciences Inner Mongolia University Hohhot People's Republic of China; ^2^ National Engineering Laboratory of Crop Stress Resistance Breeding Anhui Agricultural University Hefei People's Republic of China; ^3^ The Key Laboratory of Industrial Biotechnology, Ministry of Education, School of Biotechnology Jiangnan University Wuxi People's Republic of China

## Abstract

Soil microbial communities were investigated under the volcanic extreme environment. Soil bacterial networks exhibited higher stability than fungal networks. Holocene granite had a more complex microbial network than basalt. Soil pH and total protein were key drivers of microbial network stability.

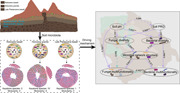

Volcanic activity is of great significance not only because it is a powerful strength shaping the global landscape but it is also a common cause of geological and ecological disturbances [1, 2]. The Wulanhada volcanoes in Inner Mongolia in Northern China erupted during the Quaternary period, during which volcanic activity could be divided into the late Pleistocene and the Holocene stages [3, 4]. Volcanic rocks provide information regarding volcanic–tectonic processes and certain tectonic environments from specific volcanic rock assemblages [3]. The volcano in Wulanhada consists dominantly of alkali basalts with a small amount of granite [5]. Volcanic eruptions lead to the destruction of surface vegetation and the loss of primary soil; therefore, soil microorganisms tend to search for habitats on rocks [6, 7]. Previous studies have shown that the colonization of rocks by microbes was influenced by the physicochemical properties of rocks, such as mineral composition, permeability, pore structure, and environmental factors, including water availability and nutrient sources [8, 9]. Numerous research on microbes has progressed in different localities and rocks. Despite the type of rocks, the colonization of microorganisms can occur in different sedimentary rocks, such as limestone [10], halite [11], gypsum [12], and igneous rocks, such as basalt and granite [13, 14]. However, the microorganisms in the rock surface soil of the volcanic extreme environment had not been widely reported, which had attracted our attention.

Previous studies have shown that some volcanic areas are extreme environments with dry atmosphere, extremely low temperature, low nutrient utilization, and high ultraviolet radiation [15, 16]. Wulanhada volcanic field belongs to the semi‐arid continental monsoon climate in the middle temperate zone, which is characterized by drought, cold wind, and large temperature differences [17]. Thus, microorganisms living in extreme environments may find suitable habitats to protect themselves [18]. The influence of extreme environments has been extensively explored. For example, in the extremely dry Atacama Desert, the denitrification function of the soil was maintained despite the microbial population being reduced [19]. Soil microbial communities can adapt to and resist short‐term temperature perturbations in cold Antarctic environments [20]. Studies have shown that unique microbial communities in extreme environments influence climate regulation, soil fertility, and ecosystem stability [21]. Soil microbes are extremely adaptable and sensitive to external environmental changes, which play an important role in maintaining ecosystem functions [22, 23]. Performing microbial co‐occurrence ecological network analyses can enhance our understanding of complex microbial communities [24, 25]. According to previous studies, various environmental factors, such as soil pH, protein, and nutrients, can affect the microbial community structure, especially in extreme environments [26]. Soil pH is the main abiotic element in shaping the soil microflora [27]. The pattern of distribution holds for entire microbial communities as well as individual microbiomes [28, 29]. In addition, soil bacterial and fungal communities have been shown to exhibit large and significant differences in the stability of certain environmental conditions [30, 31].

As mentioned above, curious about the variations of microbial communities in different rock surface soils under the volcanic extreme environment, we studied the variations of microbial communities in different rock surface soils of the Wulanhada Quaternary volcano [32]. This present study intended to further (1) identify the variations of microbial community structure in surface soils of different volcanic rocks, (2) explore major members of the fungal and bacterial communities that inhabit rocky surface soils in volcanic extreme environments, and (3) investigate the influence of various environmental factors on soil microbial communities. This research can provide new opinions for studying soil microbial communities in the extreme environment of Wulanhada Volcano.

## RESULTS

### Overview of high‐throughput sequencing and microbial community composition

**Figure 1:**
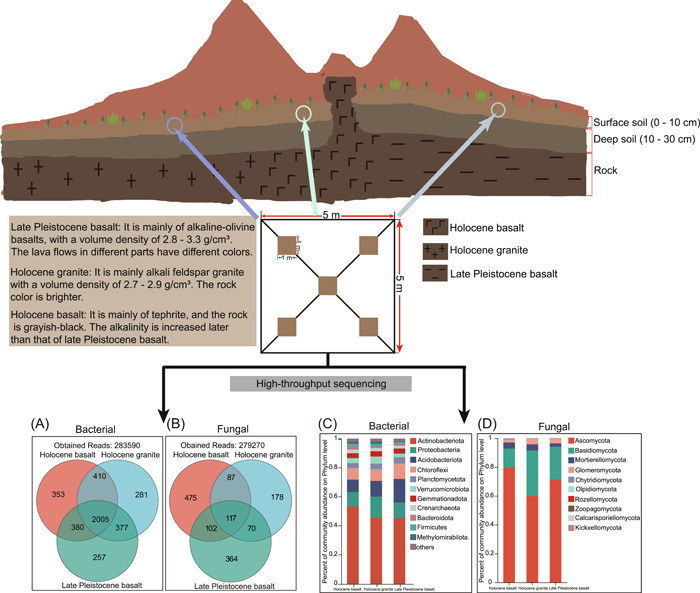
High‐throughput sequencing and community composition based on soil microbial community. The shared and unique operational taxonomic units (OTUs) of bacterial (A) and fungal (B) communities among different treatments as assessed by Venn diagram. The relative abundance of bacteria (C) and fungi (D) in different samples.

The results of soil samples subjected to Illumina paired‐end sequencing showed that the total reads of bacterial 16 S rRNA gene sequences was 447,762, with an average length of 253.10 bp. While the total reads of fungal internal transcribed spacer  gene sequence was 472,558, with an average length of 242.49 bp. Then operational taxonomic units (OTUs) belonging to the mitochondrial, chloroplast, chlorophyte were removed, and then subsampled to the minimized reads sample, namely 283,590 (bacteria) and 279,270 (fungi) reads (Figure 1A,B). The richness of soil microbial communities was assessed using sparse curves between plots based on the OTUs observed in each plot. The rarefaction curves of all the samples were saturated, suggesting that the sequencing depth was adequate and could represent almost all bacterial and fungal communities (Supporting Information: Figure S1). The rarefaction curve and principal component analysis result showed that our grouping was reliable (Supporting Information: Figure S2). The composition of the microbial community was different in different rock surface soils and different periods of the same rock surface soil (Figure 1C,D). Bacterial communities in all groups primarily comprised the phyla Actinobacteria (46.70%–52.90%), Proteobacteria (13.63%–16.10%), and Acidobacteria (10.12%–12.23%) (Figure 1C). While fungal communities in all groups primarily comprised of the phyla Ascomycota (58.30%–79.21%) and Basidiomycota (26.21%–12.78%) (Figure 1D).

### Analysis of microbial community difference and co‐occurrence network

**Figure 2:**
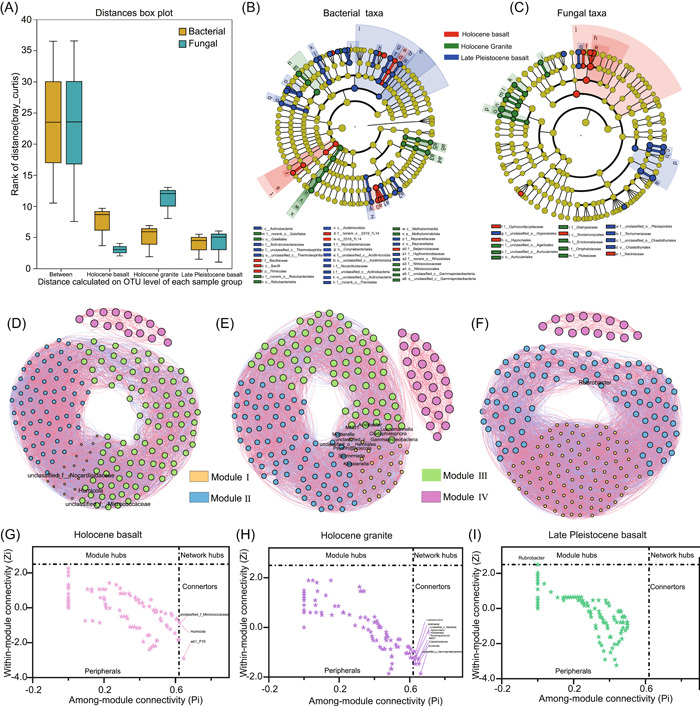
Microbial community differences and network analysis. (A) The similarity analysis (ANOSIM) of bacteria and fungi based on bray_curtis distance. The cladogram with a linear discriminant analysis (LDA) score > 3.0 representing the phylogeny of bacterial (B) and fungal (C) lineages among the three plots. The letters p, c, o, f, and g represented phylum, class, order, family, and genus, respectively. (D–F) The co‐occurrence networks of core microbial communities in three plots. (G–I) The *Zi*‐*Pi* diagram showing fungal and bacterial node distributions in the three volcanic rocks based on *Zi* (connectivity within modules) and *Pi* (connectivity between modules).

Analysis of similarity (ANOSIM) results showed that the soil bacterial community was more stable than the fungal community. (Figure 2A). The microbial communities in the three plots identified using the linear discriminant analysis effect size (LEfSe) method and the core microbial communities with statistical significance were illustrated in Figure 2B,C. The letters p, c, o, f, and g represented phylum, class, order, family, and genus, respectively. The LEfSe results revealed 63 clades (2 phyla, 4 classes, 10 orders, 17 families, and 30 genera) in the bacterial community (Figure 2B) and 32 clades (1 class, 3 orders, 12 families, and 16 genera) in the fungal community (Figure 2C). In addition, linear discriminant analysis (LDA) results showed that there were 33, 15, and 15 bacteria in the microflora of Holocene basalt, granite, and late Pleistocene basalt, respectively. For fungi, 11, 13, and 8 fungal phylotypes were detected in the microflora of Holocene basalt, granite and late Pleistocene basalt, respectively (Supporting Information: Figure S3). It shows that the composition of bacteria and fungi is different from each other, with bacteria having more biomarkers than fungi in the surface soil of different rocks.

Regarding the soil microorganisms, we previously defined the top 100 most abundant bacterial and fungal genera in all samples as the core microbiome [33, 34]. The co‐occurrence network analysis of the top 100 bacteria and fungi genera in the core soil microbial communities were used to construct the whole microbial network (Figure 2D–F) [35]. The co‐occurrence network was divided by modules, where the Holocene basalt and Holocene granite had four modules; however, the Late Pleistocene basalt had three modules. The network topology (Supporting Information: Table S2) showed that the nodes of the three plots were the same; but the total links, average connectivity (avgK), and average clustering coefficient (avgCC) were the highest in the Late Pleistocene basalt. While the average path length (GD) was the highest in the Holocene basalt. The proportion of positive correlation was greater than that of negative correlation in the co‐occurrence networks of the three plots. In contrast, the modularity (M), GD, and avgCC of the random networks were lower than the empirical networks.

Given that network hubs, connectors, and module hubs are considered keystone taxa, the nodes were divided into network hubs, peripherals, module hubs, and connectors based on the value of among‐module connectivity (*Pi*) and within‐module connectivity (*Zi*) (Figure 2G–I); network hubs, connectors, and module hubs are considered keystone taxa. The keystone taxa accessorial with the bacterial genera *unclassified_f_Micrococcaceae*, *Humicola*, and *wb1_P19* were identified in Holocene basalt (Figure 2G); *Luedemannella*, *Acidibacter*, *unclassified_o_Helotiales*, *Sporormiella*, *Keissleriella*, *Psychroglaciecola*, *MND1*, *Cladophialophora*, *Mortierella*, and *unclassified_o_Gammaproteobacteria* were identified in Holocene granite (Figure 2H); while *Rubrobacter* was identified in Late Pleistocene basalt (Figure 2I).

### Influence of environmental factors and microbial community composition on microbial network structure and fungal multifunctionality

**Figure 3:**
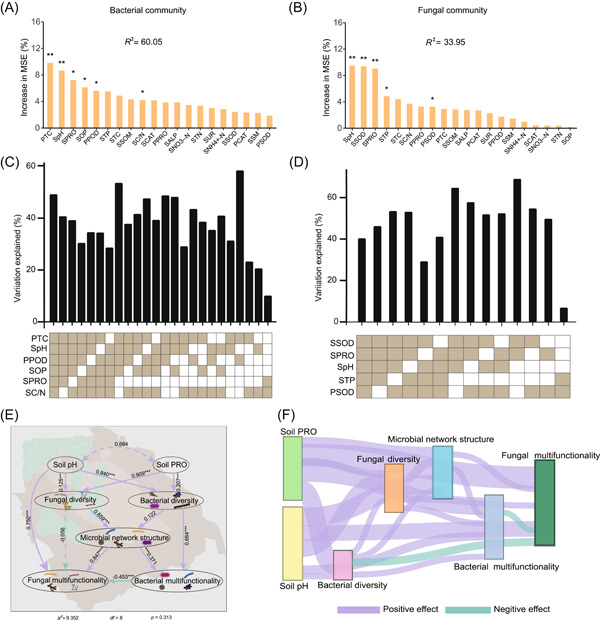
The influence of environmental factors on microbial community and the causal relationship of each attribute. (A–D) The predicted effects of different environmental factors on soil bacterial and fungal diversity based on random forest analysis. (E) The structural equation models (SEMs) showing the effects of key environmental factors on soil microbial communities. (F) The Sankey diagram based on SEM, where the thickness of the streamline represents the standard total effect size (direct plus indirect effects).

According to the full random forest model, the variations in bacterial and fungal diversity (Shannon diversity index) were 60.05% and 33.95%, respectively. Six key environmental predictors were identified in the bacterial community, with the major predictors being plant total chlorophyll content (plant TC) (9.8%), followed by soil pH (8.7%), soil total protein (soil PRO) (6.9%), soil organic phosphorus (soil OP) (7.2%), plant peroxidase (plant POD) (6.1%), and soil carbon‐to‐nitrogen ratios (soil C/N) (4.3%) (Figure 3A). Ten‐fold cross‐validation revealed that the cross‐validation error was minimal when four variables were included in the model (Supporting Information: Figure S4a). The exclusion of soil PRO and soil C/N had the greatest impact on the model accuracy, while the exclusion of Soil pH and soil PRO had the least impact on the model accuracy (Figure 3C). Additionally, five key environmental predictors were identified in the fungal community, with the major predictors being soil pH (9.6%), soil superoxide dismutase (soil SOD) (9.5%), soil PRO (8.9%), soil total phosphorus (soil TP) (5.7%), and plant superoxide dismutase (plant SOD) (3.4%) (Figure 3B). Ten‐fold cross‐validation revealed that the cross‐validation error was minimal when five variables were included in the model (Supporting Information: Figure S4b). The exclusion of soil PRO had the greatest impact on model accuracy, while the exclusion of soil pH had the least impact on model accuracy (Figure 3D). Thus, random forest analysis showed that soil microbial diversity was closely associated with soil pH and soil PRO.

Structural equation model (SEM) revealed direct and indirect relationships between key environmental factors and community composition structure, and between microbial network structure and multifunctionality (Figure 3E). Based on the results of the random forests, soil pH and soil PRO were used as the key environmental factors. Soil pH and soil PRO had a significant positive effect on bacterial and fungal community diversity. Fungal diversity had a significant positive impact on microbial network structure, while microbial network structure had a significant positive impact on fungal multifunctionality. Furthermore, microbial network structure had a significant positive impact on bacterial multifunctionality and bacterial multifunctionality had a significant negative impact on fungal multifunctionality. Soil pH had a significant positive impact on fungal multifunctionality. Additionally, the Sankey diagram showed that soil pH was a major contributor to fungal multifunctionality and fungal diversity contributed most to the microbial network structure (Figure 3F).

## DISCUSSION

Although there were some differences in the microbial community composition of the surface soil at different times in the same type of rock, the variation was not as large as that between different types of rocks. The relative abundance of Actinobacteria and Ascomycota in the surface soil of Holocene and Late Pleistocene basalts was higher than that of granite, but the degree of difference between basalt and granite was greater than that of Holocene and Late Pleistocene (Figure 1C,D). These differences may be caused by differences in rock chemical composition and the microenvironment in which they are located. Previous studies have shown that the chemical properties of different types of rocks can affect the microbial community [16]. Interestingly, the microbial community structure in granite significantly differs from that in other rock types due to the type of mineral inclusions [36]. Basalt has a higher magnesium content than other rock types [16]. The connectivity of microbial communities in the surface soil of Holocene granite was better than that of Holocene basalt, with higher avgCC and avgK (Figure 2D–F). Furthermore, keystone species are often closely related taxa that considerably impact microbial networks [37]. Studies have shown that key species in natural ecosystems often exhibit variations after succession, which substantially influences the biogeochemical cycling of nutrients, such as carbon, nitrogen, and phosphorus, in addition to regulating ecosystem functions [38, 39]. Different rock types also have different key species, with the most key species in the surface soil of granite. It shows that the properties of granite may significantly influence the microbial community in the surface soil, which this result was few reported in previous research and requires for further investigation. Therefore, we speculate that the composition of the rock may affect the microbial community in the soil covering the rock surface. For the same number of nodes, the Late Pleistocene basalt had more connections, higher avgK, and higher avgCC than that of the Holocene basalt. This is consistent with previous research showing that microbial communities are more structurally diverse in older volcanic soils than in younger volcanic soils [40].

The archaea were very low in this study (1%–2%), hence only bacterial and fungal were analyses and discussed subsequently. The Actinobacteria, Proteobacteria, Acidobacteria, Ascomycota, and Basidiomycota were dominant in all soil samples. Members of these phyla have been found to survive well in extreme environments [41]. Volcanoes are extreme environments and microorganisms that inhabit the surface soil in these areas need to adapt to the existing conditions. Previous studies have shown that the magnesium content of basalt is higher than in other rock types [14, 16], and magnesium affects the distribution of Actinobacteria communities in cold environments [42]. Members of Actinobacteriota can produce spores that withstand extreme environments and promote rock weathering [43]. Proteobacteria show lower abundance than Actinobacteria (Figure 1), but they are pro‐colonizers commonly found in nutritionally endowed environments and grow under environments with a high organic substrate content [44]. Previous studies have explained that rapid‐growth bacteria prefer nutritionally endowed environments, while tardy‐growth oligotrophic bacteria can survive in low nutritional environments [16, 45, 46]. Similar to these research, the low abundance of Proteobacteria may be attributed to the lack of nutrients in the extreme environment of the Wulanhada volcanic.

Soils developed from various rocks have varying physicochemical properties, which strongly influence the bacterial and fungal communities [47]. Microorganisms form close links with plants to adapt to different environmental conditions [48, 49]. Plant TC was the major factor affecting bacterial diversity (Figure 3A), and chlorophyll derivatives are radical quenchers [50, 51]. According to a previous study, radical quenchers exert irreversible effects on microorganisms [52]. Interestingly, in this study, soil pH was identified as a key factor affecting fungal diversity (Figure 3B). Soil pH has been shown to be a primary driver of the distribution of soil microbial communities [53]. Soil pH regulates enzyme activities in the soil, mineralization of organic matter, and biodegradation [54]. Notably, the interactive effect of plant and soil factors was stronger than either single factor, suggesting that soil microbial communities in volcanic rocks were influenced by multiple factors. SEM is an a priori method for visualizing causal relationships between variables, primarily by fitting data to a causal hypothesis model [55]. SEM revealed the direct and indirect relationships between key environmental factors and community composition structure, and between microbial network structure and multifunctionality, which further supports the crucial effect of soil pH on microbial communities (Figure 3E). Notably, fungal alpha diversity was a major contributor to the microbial network structure, while soil pH was a major contributor to fungal functions, which is consistent with previous studies, suggesting that fungi are more resilient to extreme environments [22, 39, 56]. Since the temperature has a great effect on the microbial community, more soil samples and experiments should be carried out in the future to explore the effect of rocks on the distribution of the soil microbial community in the volcanic environment.

## CONCLUSIONS

This study investigated the composition and diversity of microbial communities in the surface soil of different rocks in the Wulanhada volcanic field. According to the results, there were significant differences in the composition of microbial communities of the three rocky surface soils; and the soil bacterial communities were more stable than the fungal communities. In addition, our results showed that soil PH and total protein had significant effects on soil microbial communities in the three volcanic rocks. These findings are of great significance in enhancing our comprehension on the distribution of the soil microbial community in volcanic environments and the contribution of environmental factors.

## AUTHOR CONTRIBUTIONS

Jin Chen and Zishan Li conceived the study, conducted data analysis, and wrote the manuscript. Qingchen Xiao and Xiaoyu Li analyzed the data. Daolong Xu, Haijing Liu, Lumeng Chao, Hanting Qu, Yaxin Zheng, Xinyan Liu, and Pengfei Wang designed and carried out the experiments. Yuying Bao supervised the whole process. All authors have read and approved the final manuscript.

## CONFLICT OF INTEREST STATEMENT

The authors declare no conflict of interest.

## Supporting information

Supporting information.

Supporting information.

## Data Availability

All the sequencing data have been deposited in NCBI Sequence Read Archive (SRA) under accession number PRJNA911355 (https://www.ncbi.nlm.nih.gov/bioproject/PRJNA911355) and PRJNA911411 (https://www.ncbi.nlm.nih.gov/bioproject/PRJNA911411). Supplementary materials (methods, figures, tables, scripts, graphical abstract, slides, videos, Chinese translated version and update materials) may be found in the online DOI or iMeta Science http://www.imeta.science/.
