# Supplementary material for: Patterns and drivers of microbiome in different rock surface soil under the volcanic extreme environment
Source: Imeta. 2023 Jun 19;2(3):e122. doi: 10.1002/imt2.122 (PMC10989942; doi:10.1002/imt2.122)
Supplement: Supplementary file 1 — Supporting information. [file IMT2-2-e122-s001.docx]

**Supplemental material**

**Patterns and drivers of microbiome in different rock surface soil under the volcanic extreme environment**

Running title：Microbiome in different rock surface soil

Jin Chen^§, 1, 2^, Zishan Li^§, 2^, Daolong Xu ^3^, Qingchen Xiao ^2^, Haijing Liu^1^, Xiaoyu Li^2^, Lumeng Chao^1^, Hanting Qu^1^, Yaxin Zheng^1^, Xinyan Liu^1^, Pengfei Wang^1^, Yuying Bao^1 *^

1. Key Laboratory of Forage and Endemic Crop Biotechnology, Ministry of Education, School of Life Sciences, Inner Mongolia University, Hohhot, P. R. China.
2. National Engineering Laboratory of Crop Stress Resistance Breeding, Anhui Agricultural University, Hefei, P. R. China.

3. The Key Laboratory of Industrial Biotechnology, Ministry of Education, School of Biotechnology, Jiangnan University, Wuxi, P. R. China.
^§^Author Contributions: Jin Chen. and Zishan Li contributed equally to this work.

*Corresponding author Address: Inner Mongolia University, 235 West University Rd, Hohhot P. R. China.

Tel/Fax: +86 471 4492944

E-mail address: [ndbyy@imu.edu.cn](mailto:ndbyy@imu.edu.cn) (Yuying Bao)

**Supplementary materials and methods**

**Sampling site**

The sampling site is located in the Wulanhada volcanic field (113°02′–113°15′E, 41°30′–41°40′N) in Inner Mongolia Autonomous Region, North China. The area has a mid-temperate semiarid continental monsoon climate. The mean annual precipitation is 292 mm and the mean annual temperature is 3.4 °C. The Wulanhada Quaternary volcano has several different rock layers distributed on its surface and the soil type is Calcic-orthic Aridisols, with the dominant vegetation in the area being shrubs and herbages. The topsoil of three types of rock layers namely Holocene basalt, Holocene granite, and Late Pleistocene basalt were independently collected for this study (Table S1).

**Sample collection and physicochemical properties**

Volcanoes are composed of different rocks, and the volcanic ash is a unique parent material for soils. Over time, microorganisms and plants colonized on the surface of the rock enhance rock weathering and soil organic matter accumulation, thus forming about 20-30cm of soil. Our study is based on this soil as the research object [1,2,3]. Based on the five-point sampling method, each sample plot was randomly set up with a large 5 m × 5 m sample square and five small 1 m × 1 m sample squares at the four corners and the diagonal center of the large sample square [4]. A total of five soil samples (1 m × 1 m) were collected in each sampling plot and then thoroughly mixed together to form a single composite sample. Soil and plant samples were collected from the rock topsoil (0–10 cm). Each soil sample was sieved through a 2-mm mesh to remove stones, plant materials, and soil organisms. The soil samples were immediately transported to the laboratory in containers with liquid nitrogen. Subsequently, a quarter of the samples were stored at 4 °C and used to analyze soil physicochemical properties while the remaining three-quarters of the samples were stored at −20 °C for genomic DNA extraction. The physicochemical parameters of plants included plant superoxide dismutase (plant SOD), plant catalase (plant CAT), plant peroxidase (plant POD), plant proline (plant PRO), and plant total chlorophyll content (plant TC). Soil physicochemical parameters included soil total nitrogen (soil TN), soil total carbon (soil TC), soil carbon-to-nitrogen ratio (soil C/N), soil ammonium nitrogen (soil NH4^+^-N), soil superoxide dismutase (soil SOD), soil total protein (soil PRO), soil catalase (soil CAT), soil moisture (soil SM), soil pH, soil alkaline phosphatase (soil ALP), soil urease (soil UR), soil nitrate-nitrogen (soil NO_3_^-^-N), soil organic matter (soil SOM), soil total phosphorus (soil TP), and soil organic phosphorus (soil OP). First, superoxide anion (O2-.) was produced through a reaction between xanthine and xanthine oxidase in a reaction system. Nitroblue tetrazolium (WST-8) was reduced to produce blue fuel formazan, which was used to determine the contents of plant SOD and soil SOD by ultraviolet spectrophotometry. Plant CAT, soil CAT, and plant TC contents were measured by ultraviolet spectrophotometry. Plant plant POD can catalyze hydrogen peroxide to oxidize the substrate o-methyl phenol to produce a tawny product, which is then measured by ultraviolet spectrophotometry. We used salicylic acid and ultraviolet spectrophotometry to determine plant proline plant PRO content. Soil soil TN and soil soil TC were determined using an elemental analyzer (Vario MACRO cube; Elementar Analysensysteme GmbH, Hanau, Germany), and the values obtained were used to calculate soil C/N. Soil TP and soil OP concentrations were determined by the molybdenum blue method. Soil NH4+-N and soil NO3--N were determined by a continuous flow analysis system. Soil PRO in acidic environments was measured using Coomassie Brilliant Blue G-250 and ultraviolet spectrophotometry. Soil SM was determined by the gravimetric method. Soil pH was measured in a 1:5 (w/v) water–soil mixture using a combination electrode. Soil ALP and soil UR activities were determined by colorimetric method. Soil OM was measured by potassium dichromate oxidation spectrophotometry.

**DNA extraction, manipulation, and high-throughput sequencing**

The extraction of genomic DNA from 0.4 g of soil (in triplicate) was performed using the PowerSoil DNA Isolation Kit (MO BIO Laboratories, Carlsbad, CA, USA), according to the manufacturer's instructions. The concentration and purity of the DNA samples were measured using a NanoDrop 2000 (NanoDrop Technologies Inc., Wilmington, DE, USA). The V4 hypervariable region of the bacteria 16S rRNA gene [5] was amplified with primer pairs 515 forward (5′ − GTGCCAGCMGCCGCGGTAA −3′) and 806 reverse (5′ − GGACTACHVGGGTWTCTAAT −3′). Fungal internal transcribed spacer (ITS) genes were amplified using the primers ITS1 forward (5′− CTTGGTCATTTAGAGGAAGTAA −3′) and ITS2 reverse (5′ − GCTGCGTTCTTCATCGATGC−3′) (Adams et al., 2013). The polymerase chain reaction (PCR) mixture (25 μL) contained 30 ng of soil genomic DNA, 4 μL (2 ng/μL) of bovine serum albumin, 0.1 μL (5 μM) of each primer, 12.5 μL of 2 × Taq Plus Master Mix (TransGen Biotech Co., Ltd., Beijing, China), as described by Chen et al. [22]. The PCR amplification of the 16S rRNA gene was performed as follows: an initial hot-start denaturation for 5 min at 94 °C, followed by 27 cycles of denaturation for 30 s at 94 °C, annealing for 30 s at 55 °C, with an extension for 60 s at 72 °C, and a final extension for 7 min at 72 °C. Afterward, the PCR products were stored at 4 °C. The PCR amplification of the fungal ITS rRNA gene region was conducted with an initial denaturation at 95 °C for 90 s; 35 cycles of 95 °C for 30 s, 55 °C for 30 s, and 72 °C for 45 s; and a final extension at 72 °C for 10 min. The PCR products were electrophoresed on 2% agarose gel and purified using an AxyPrep DNA Gel Extraction Kit (Axygen Biosciences, Union City, CA, USA) to eliminate contaminants.

After quantification using a NanoDrop® ND-1000 spectrophotometer (NanoDrop® Technologies, Wilmington, DE, USA), equimolar amounts of amplicons were pooled before sequencing on an Illumina MiSeq system (Illumina, San Diego, CA, USA) at ALLWEGENE Inc. (Beijing, China). The raw sequences were re-assigned according to their barcodes and were quality-trimmed using Mothur 1.32.2 [6,7], where chimeric sequences were eliminated. Then, the non-chimeric sequences were clustered into operational taxonomic units (OTUs) using the UPARSE-pipeline, setting a distance limit of 0.03 (equivalent to 97% similarity) using the open-reference OTU picking protocol [8]. A representative sequence was aligned using the Python Nearest Alignment Space Termination (PyNAST) against sequences within the SILVA database for bacteria and the Unite database for fungi. The effects of sampling on diversity were corrected by rarifying the sequence numbers of each sample to that of the sample with the lowest number of reads, after which the microbial diversity, microbial richness, and sequencing depth in each sample was estimated with the Shannon, Chao and Good’s Coverage indices using Mothur (http://www.mothur.org/).

The Illumina MiSeq sequencing data for bacteria and fungi were deposited in the Sequence Read Archive (SRA) at the National Center for Biotechnology Information (NCBI) under the accession numbers PRJNA911355 and PRJNA911411, respectively.

**Statistical analyses**

The unique and shared OTUs among the three plots and the relative abundance of microbial community phylum were visualized by “ggplot2” package in R software. Similarity analysis (ANOSIM) based on Bray-Curtis distance showed differences in soil bacterial and fungal communities. The linear discriminant analysis effect size (LEfSe) method was used to identify biomarkers of each microbial group at multiple taxonomical levels, with a linear discriminant analysis (LDA) effect size threshold of 3.0 and alpha value threshold of 0.05 for the factorial Kruskal–Wallis test (http://huttenhower.sph.harvard.edu/galaxy)**.** The co-occurrence networks of the core soil microbial communities were constructed using iNAP (*P* < 0.01, Spearman correlations with *r* > 0.8) (<http://mem.rcees.ac.cn:8081/>) [9−11]. The networks were visualized using Gephi software. The properties of nodes within-module connectivity (*Zi*) and among-module connectivity (*Pi*) were used to identify key flora in the network, which included network hubs (*Zi* > 0.25, *Pi* > 0.62), connectors (*Zi* ≤ 0.25, *Pi* > 0.62), and module hubs (*Zi* > 0.25, *Pi* ≤ 0.62). In order to evaluate the relative importance of measurement variables, a random forest model was constructed using the “randomForest” and “ggplot2” packages. All environmental factors were used to develop a full random forest model, and the significant variables identified in the full model were used to develop more accurate sparse the importance of an individual variable was compared using the increase in mean squared error (IncMSE) of the model and the significance of IncMSE when it was the randomly replaced. The significant variables were then used to develop a more accurate model. Before the construction of the sparse model, 10-fold cross validation was performed using the “ggplot2” package to determine how many variables should by induced [12]. Structural equation models (SEMs) were developed using IBM SPSS Statistics 26.0 and IBM SPSS Amos 26.0 Graphics (IBM Corporation, Armonk, NY, USA) [13]. The Sankey diagram was constructed using the “networkD3” package in RStudio.

**References**

1. Bai, Zhida, Jianming Wang, Guiling Xu, Lei Liu, and Debin Xu. 2008. “Quaternary Volcano Cluster of Wulanhada, Right-back-banner, Chahaer, Inner Mongolia.” *Acta Petrologica Sinica* 24(11): 2585−2594. <http://doi.org/1000-0569/2008/024(11)-2585-94>

2. Dubroeucq, Didier, Daniel Geissert, and Paul Quantin. 1998. “Weathering and soil forming processes under semi-arid conditions in two Mexican volcanic ash soils.” *Geoderma* 86(1-2), 99−122. <https://doi.org/10.1016/S0016-7061(98)00033-0>.

3. Hernández, Marcela, Marcela Calabi, Ralf Conrad, and Marc G. Dumont. 2020. “Analysis of the microbial communities in soils of different ages following volcanic eruptions.” *Pedosphere* 30(1), 126−134. <https://doi.org/10.1016/S1002-0160(19)60823-4>4. Gao, Meixiang, Ping He, Xueping Zhang, Dong Liu, and Donghui Wu. 2014. “Relative roles of spatial factors, environmental filtering and biotic interactions in fine-scale structuring of a soil mite community.” *Soil Biology and Biochemistry* 79: 68−77. <https://doi.org/10.1016/j.soilbio.2014.09.003>

5. Zhang Junyi, Xiao Ding, Rui Guan, Congmin Zhu, et al. 2018 “Evaluation of different 16S rRNA gene V regions for exploring bacterial diversity in a eutrophic freshwater lake.” *Science of the Total Environment* 618: 1254−1267. <https://doi.org/10.1016/j.scitotenv.2017.09.228>

6. Schloss, Patrick D, Sarah L. Westcott, Thomas Ryabin, Justine R. Hall, Martin Hartmann, Emily B. Hollister, Ryan A. Lesniewski, Carolyn F. Weber, et al. 2009. “Weber Introducing mothur: Open-source, platform-independent, community-supported software for describing and comparing microbial communities” *Appl. Environ. Microbiol* 75 (2009), pp. 7537−7541 <https://doi.org/10.1128/AEM.01541-09>

7. Li, Wenjun, Likun Wang, Xiaofang Li, Xin Zheng, Michael F. Cohen, Yong-Xin Liu, et al. 2022. “Sequence-based Functional Metagenomics Reveals Novel Natural Diversity of Functioning CopA in Environmental Microbiomes.” *Genomics, Proteomics & Bioinformatics* 20: 2022.2008.2006. <https://doi.org/10.1016/j.gpb.2022.08.006>

8. Robert, C Edgar. 2013. “UPARSE: highly accurate OTU sequences from microbial amplicon reads.” *Nature methods* 10(10), 996−998. <https://doi.org/10.1038/nmeth.2604>

9. Feng, Kai, Xi Peng, Zheng Zhang, Songsong Gu, Qing He, Wenli Shen, Zhujun Wang, Danrui Wang, Qiulong Hu, Yan Li, Shang Wang, and Ye Deng. 2022 “iNAP: an integrated Network Analysis Pipeline for microbiome studies.” *iMeta*. 1: e13. <https://doi.org/10.1002/imt2.13>

10. Tong, Chen, Yong-Xin Liu, Luqi Huang. 2022. “ImageGP: An easy-to-use data visualization web server for scientific researchers.” *iMeta* 1: e5. <https://doi.org/10.1002/imt2.5>

11. Wen, Tao, Penghao Xie, Shengdie Yang, Guoqing Niu, Xiaoyu Liu, Zhexu Ding, Chao Xue, Yong-Xin Liu, Qirong Shen, and Jun Yuan. 2022. “ggClusterNet: An R package for microbiome network analysis and modularity-based multiple network layouts.” *iMeta* 1: e32. <https://doi.org/10.1002/imt2.32>

12. Wei Xiaomeng, Yajun Hu, Guan Cai, Huaiying Yao, Jun Ye, Qi Sun, Stavros Veresoglou, rt al. 2021. “Organic phosphorus availability shapes the diversity of phoD-harboring bacteria in agricultural soil.” *Soil Biology and Biochemistry.* 161, 108364. <https://doi.org/10.1016/j.soilbio.2021.108364>

13. Wei Yuquan, Hao Zhang, Ying Yuan, Yingshuang Zhao, Guanghe Li, and Fang Zhang. 2020. “Indirect effect of nutrient accumulation intensified toxicity risk of metals in sediments from urban river network.” *Environmental Science and Pollution Research.* 27(6), 6193−6204. <https://doi.org/10.1007/s11356-019-07335-9>

**Supplementary figure legends**

**Figure S1**. Shannon diversity indices and readings for OTUs were used to construct dilution curves of the sequence reads for bacterial (A) and fungal (B) communities in the three volcanic rock soils.

**Figure S2** Principal component analysis of the composition of bacterial (A) and fungal (B) communities in the different plots.

**Figure S3** Linear discriminant analysis (LDA) of community sizes of bacterial (A) and fungal (B) community size in the three types of volcanic rocks.

**Figure S4**. Ten-fold cross-validation errors for bacterial (A) and fungal (B) diversity in the soil, respectively. The error increased with an increase in environmental factors.

**Table S1:** Basic characteristics of the three types of sites.

**Table S2:** Topological properties of the empirical ecological networks (MENs) of core microbiomes.


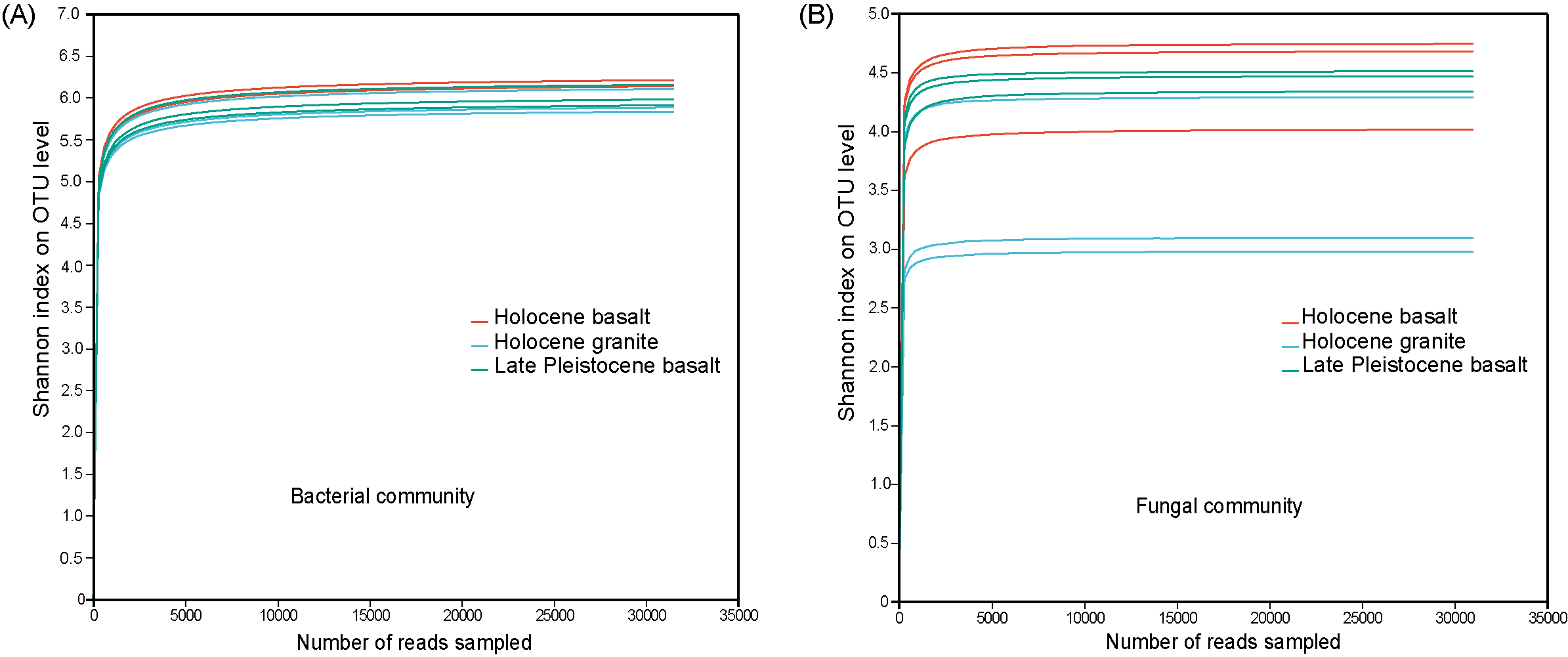


**Figure S1**. Shannon diversity indices and readings for OTUs were used to construct dilution curves of the sequence reads for bacterial (A) and fungal (B) communities in the three volcanic rock soils.


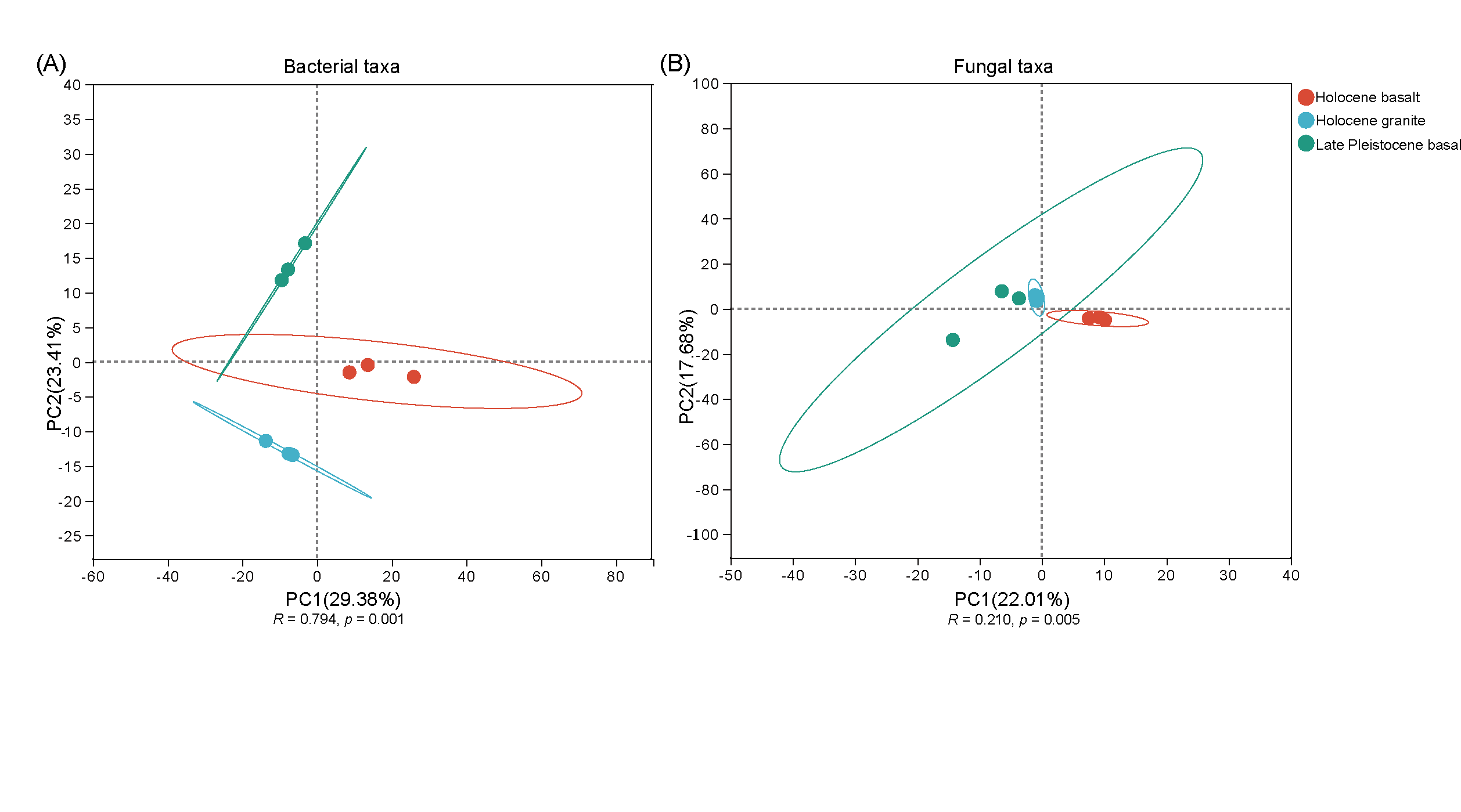
**Figure S2** Principal component analysis of the composition of bacterial (A) and fungal (B) communities in the different plots.


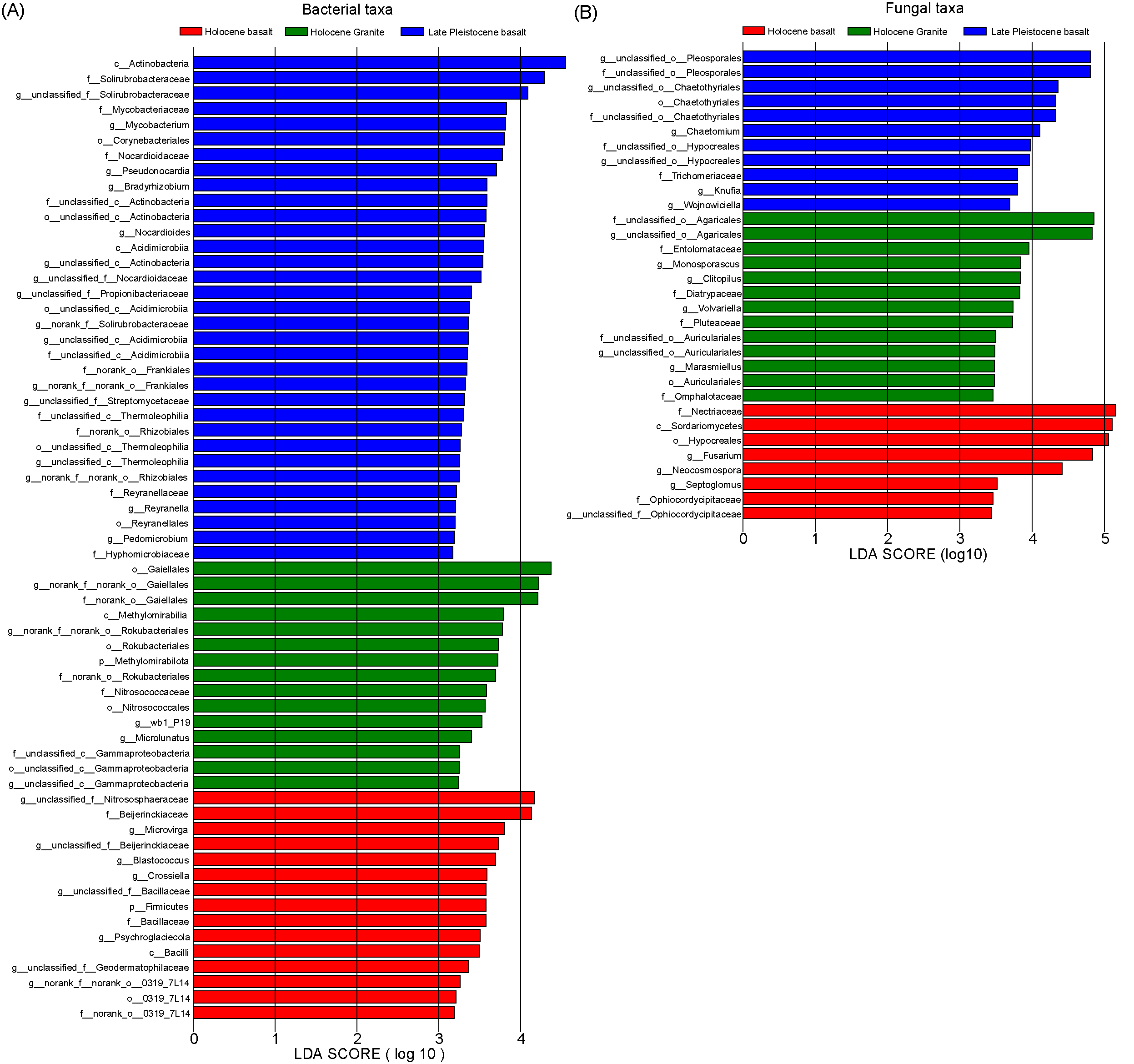


**Figure S3** Linear discriminant analysis (LDA) of community sizes of bacterial (A) and fungal (B) community size in the three types of volcanic rocks.


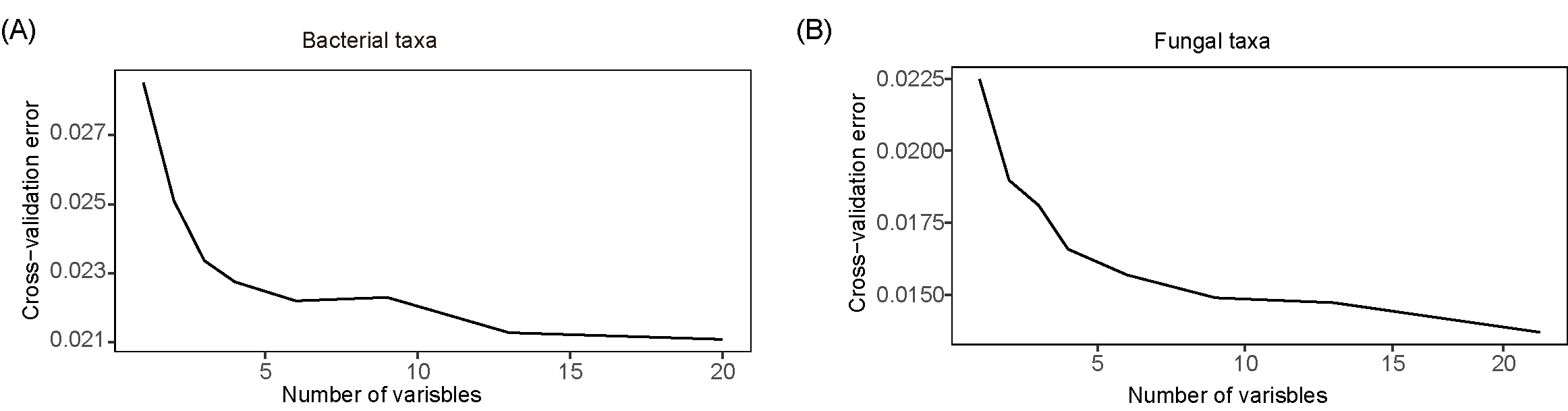


**Figure S4**. Ten-fold cross-validation errors for bacterial (A) and fungal (B) diversity in the soil, respectively. The error increased with an increase in environmental factors.
